# Supplementary material for: Nutrition and Sensory Evaluation of Solid-State Fermented Brown Rice Based on Cluster and Principal Component Analysis
Source: Foods. 2022 May 25;11(11):1560. doi: 10.3390/foods11111560 (PMC9180828; doi:10.3390/foods11111560)
Supplement: Supplementary file 1 [file foods-11-01560-s001.zip › foods-1699633-supplementary.pdf]

Supplementary

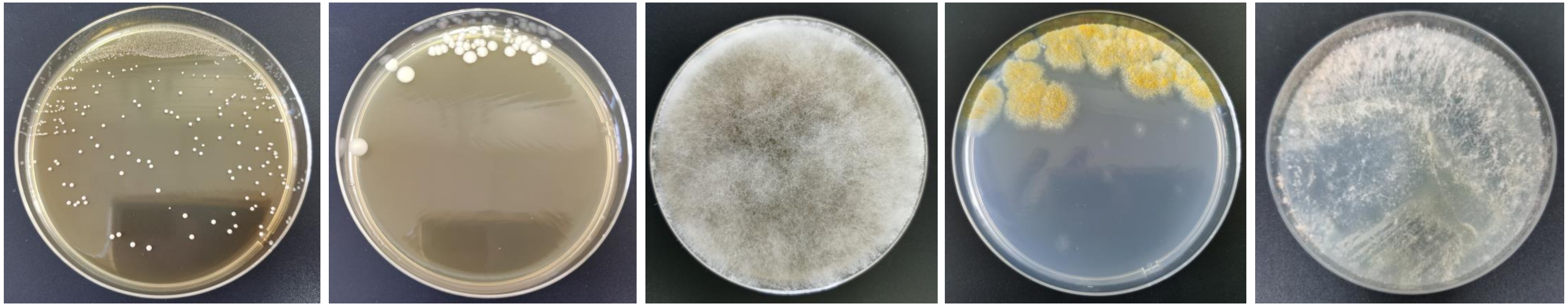

L. plantarum ***S. cerevisiae*** ***R. oryzae*** ***A. oryzae*** ***N. sitophila***

Figure S1. The growth state of the five strains.

Table S1. Criteria for sensory scoring.

Assessment Indicator<br>Primary Index	Secondary Index	Criteria	Range
**Smell (20 points)**	Purity	Delicate rice smell with strong unique fermented savory smell, no odor distribution	15~20
		Apparent rice smell with in-apparent unique fermented savory smell, no odor distribution	12~14
		Medium rice smell without unique fermented savory smell, with certain peculiar smell	7~11
		Obvious peculiar smell	0~6
**Appearance (20 points)**	Color (10 points)	Light yellow or white with bright color	7~10
		Light yellow with general luster	4~6
		Dark yellow and dark in color	0~3
	Degree of whiteness (10 points)	Large cracking with high whiteness	7~10
		Little cracking with slight whiteness	4~6
		No cracking without no whiteness	0~3
**Taste (20 points)**	Fragrance (10 points)	Strong fragrance without peculiar smell when chewing	9-10
		Faint fragrance without peculiar smell when chewing	6-8
		No fragrance and peculiar smell when chewing	4-6
		No fragrance with obvious peculiar smell when chewing	0-3
	Taste produced by fermentation (10 points)	Obvious nice and unique sour taste featured of fermentation when chewing, no strange taste	9-10
		Weak in unique sour taste featured of fermentation when chewing, no strange taste	6-8
		No obvious unique sour taste featured of fermentation when chewing, no uncomfortable taste	4-6
		No obvious unique sour taste featured of fermentation when chewing, with obvious uncomfortable taste	0-3
**Overall acceptability (30 points)**	Viscosity or stickiness (10 points)	Clingy but not sticky	8~10
		Less clingy but not stick to teeth	5~7
		Stick to teeth or without viscosity	0~4
	Elasticity or resilience (10 points)	Muscular and chewy	8~10
		Slight muscular and chewy	5~7
		No muscular and chewy	0~4
	Hardness (10 points)	Moderate in hardness and softness	8~10
		Inclined to hard or soft	5~7
		Very hard or soft	0~4
**Texture (10 points)**	Compactness and cohesiveness (10 points)	Fluffy, uniform dispersed, without agglomeration	7~10
		Less fluffy, slight agglomerate	4~6
		Easy to bond together	0~3
